# Amino acid digestibility in diets containing copra meal with β-mannanase fed to growing pigs

**DOI:** 10.5713/ab.21.0019

**Published:** 2021-03-11

**Authors:** Jae Cheol Jang, Dong Hyuk Kim, Young Dal Jang, Yoo Yong Kim

**Affiliations:** 1 Department of Agricultural Biotechnology, and Research Institute of Agriculture and Life Sciences, Seoul National University, Seoul 08826, Korea; 2 Department of Animal and Food Science, University of Wisconsin-River Falls, River Falls, WI 54022, USA

**Keywords:** Amino Acid Digestibility, β-Mannanase, Copra Meal, Standardized Ileal Digestibility, Pigs

## Abstract

**Objective:**

The objective of this study was to estimate standardized ileal digestibility (SID) of amino acids (AA) in growing pigs fed diets containing increasing levels of copra meal (CM) with β-mannanase supplementation.

**Methods:**

Twenty barrows (initial body weight: 34.43±0.11 kg) surgically fitted with T-cannulas at the distal ileum were individually housed in metabolism crates. Pigs were allotted to 5 dietary treatments in a completely randomized design with 4 replicates per treatment. The dietary treatments were: i) NC, negative control, corn-soybean meal (SBM) based diet, ii) PC, positive control, basal diet + 0.10% β-mannanase supplementation (800 IU/kg), iii) CM6, PC diet with 6% CM supplementation, iv) CM12, PC diet with 12% CM supplementation, and v) CM18, PC diet with 18% CM supplementation. A nitrogen-free diet was used to estimate basal endogenous losses of AA for SID calculation. All experimental diets contained 0.5% chromic oxide as an indigestible marker. Each period consisted of a 4-d diet adaptation period and a 3-d ileal digesta collection period.

**Results:**

There were no differences in apparent ileal digestibility (AID) and SID of all AA between the NC and PC treatments except that the PC treatment had lower AID and SID of glycine than the NC treatment (p<0.05). There were linear decreases in AID and SID of lysine (p<0.05) and aspartic acid (p = 0.06; tendency) with increasing levels of CM in the diets with β-mannanase.

**Conclusion:**

The β-mannanase supplementation had no effect on AA digestibility in pigs fed the corn-SBM based diet but increasing levels of CM reduced SID of lysine and aspartic acid.

## INTRODUCTION

Copra meal (CM) is a by-product of oil-extraction from coconut kernel, and has been used in swine diets in the tropical regions. According to NRC [1], the CM contains 21.9% crude protein (CP), 0.58% lysine (Lys), and 0.35% methionine (Met). However, heat damage in the CM processing (such as drying, oil extraction, and range of heat) may affect nutrient availability and result in fluctuation of standardized ileal digestibility (SID) of amino acids (AA) such as SID of Lys [2], varying from 21.4% [3] to 72.8% [4]. This may cause inaccurate assessment of digestible AA content in the diet formulation that could result in variations on growth performance and nutrient utilization in pigs [5–7].

The presence of high levels of non-starch polysaccharides (NSP) in the CM have restricted their use in monogastric animal diets, because of anti-nutritional properties of NSP [8]. The major NSP in the CM is β-mannan, which constitutes 25% to 35% [9]. It has been proposed that the β-mannan in the diets can be degraded into mannose or manno-oligosaccharides by supplementation of exogenous β-mannanase [10]. Although there have been a few dedicated studies evaluating SID of AA in the CM [3,4], limited information is available for estimating the SID of AA in the diets containing increasing levels of CM with β-mannanase fed to growing pigs. It is important to investigate how the inclusion level of CM in the diets affects AA digestibility because it was reported that the CM supplementation in the swine diets reduced protein digestibility [7]. Therefore, the objective of the current study was to determine the effect of increasing levels of CM in the diets with β-mannanase fed to growing pigs on SID of AA.

## MATERIALS AND METHODS

### Animal care

Animal Experimental Guidelines provided by the Seoul National University Institutional Animal Care and Use Committee (SNU-IACUC; SNU-160613-10) approved the experimental procedure.

### Experimental design, diets and feeding

Twenty barrows ([Yorkshire×Landrace]×Duroc) with an initial body weight of 34.43±0.11 kg (stan-dard deviation = 2.7) were allocated to 5 dietary treatments in a completely randomized design with 4 replicates per treatment. The pigs were surgically fitted with a T-cannula at the distal ileum, approximately 15 cm cranial to the ileocecal junction [11]. After surgery, the pigs were housed in individual metabolic cages equipped with a feeder and a nipple drinker, and allowed to recover for 14-d before the beginning of the digesta collection.

The dietary treatments were: i) NC, negative control, a corn-soybean meal (SBM) based diet; ii) PC, positive control, basal diet + 0.10% β-mannanase (800 IU/kg); iii) CM6, PC diet with 6% CM supplementation; iv) CM12, PC diet with 12% CM supplementation; and v) CM18, PC diet with 18% CM supplementation (Table 2). The β-mannanase (800,000 U of β-mannanase/kg; Patent, 10–0,477,456–0000; CTC Bio Inc., Seoul, Korea) used in the present study was obtained from a commercial company (CTC Bio, Inc.), which has been produced by *Bacillus subtilis* (WL-7) grown on Luria broth. Each experimental diet was formulated to contain 3,265 kcal metabolizable energy (ME)/kg, 18.0% CP, 0.95% total Lys and 0.25% total Met and other nutrients were also met or exceeded NRC [12]. The CM was supplemented to each diet at the assigned level (6%, 12%, and 18%) by replacing corn and SBM, and the synthetic lysine was supplemented to meet the AA requirements. A corn starch-based N-free diet was used to estimate basal endogenous losses of AA (Table 3). All experimental diets contained 0.5% chromic oxide as an indigestible marker.

### Sample collection

During a 14-d recuperation period, all pigs were fed a common commercial growing phase diet containing 23% CP and allowed *ad libitum* access to feed and water. The experimental period consisted of a 4-d adaptation period and a 3-d collection period. The quantity of feed provided daily per pig was calculated as approximately 2.0 times the estimated maintenance energy requirement (106 kcal of ME/kg BW^0.75^) adjusted in the NRC [12] on the basis of calculated ME concentration in the diets. The feed was divided into two equal meals and fed to pigs at 0700 and 1900. Water was provided *ad libitum*. The ileal digesta were collected during 12 h from 0800 to 2100 for 3 consecutive days followed by Jorgensen et al [13]. A 200 mL of sterilized plastic bag was attached to T-cannula barrel using a cable tie. The bag was checked every 30 min and removed immediately when it exceeded a two-third level. The collected samples from each pig were placed in separate bags and stored at −80°C to prevent bacterial degradation of AA in the ileal digesta. At the end of collection phase, all collected samples were thawed, pooled, lyophilized to obtain a solid form using a freeze-dryer, and then finely ground for chemical analysis.

### Chemical analysis

Ingredient and diet samples were analyzed for dry matter (DM) by oven drying at 105°C for 3 h (method 935.29), CP (method 990.03), ether extract (EE; method 920.39), calcium (Ca; method 978.02), and phosphorus (P; method 946.06) according to AOAC [14]. Chromic oxide in ileal digesta and experimental diets was determined according to Fenton and Fenton [15]. The AA concentrations in the experimental diets and ileal digesta were analyzed using acid hydrolysis method (method 994.12) except for sulfur-containing AA (method 985.28) and tryptophan (method 988.15) followed by AOAC [14].

### Digestibility calculation

The apparent ileal digestibility (AID) of AA was calculated with chromium contents in the diets and ileal digesta by the following equation developed by Fan et al [16];

AID=(100-[(AAd/AAf)×(Crf/Crd)])×100 (%)

where AAd is the amino acid content in ileal digesta DM, AAf is the amino acid content in feed DM, and Crf and Crd are the chromium content in feed and ileal digesta DM, respectively.

The endogenous AA losses (EAL, mg/kg DM intake), induced by the N-free diet, were followed by the equation developed by Moughan et al [17];

EAL=[AAd×(Crf/Crd)]

The SID of AA was calculated based on the AID and EAL values according to the equation developed by Jondreville et al [18] as follows;

SID=AID+[EAL/AAf)×100

### Statistical analysis

Data were analyzed by analysis of variance using the GLIMMIX procedure of SAS (SAS 9.4 Inst. Inc., Cary, NC, USA) with a completely randomized design. Individual pig was used as an experimental unit. Homogeneity of variances was confirmed and outliers were tested using the UNIVARIATE procedure of SAS (SAS 9.4 Inst. Inc., USA) and no outlier was removed. A single degree of freedom contrast was performed for the comparison between the NC and PC treatments to verify the β-mannanase effect. Orthogonal polynomial contrasts were used to evaluate linear and quadratic responses with increasing levels of CM in the diets using PC, CM6, CM12, and CM18 treatments. The least squares means of each treatment were calculated and the difference in means was tested using the PDIFF option with the Tukey’s adjustment. Statistical differences were considered significant at p<0.05 and tendency at p<0.10.

## RESULTS AND DISCUSSION

### Nutrient composition of the ingredients and diets

The chemical (DM, CP, EE, Ca, and P) and AA composition of ingredients and experimental diets are provided in Tables 1 and 2, respectively. The content of CP in the CM used in this experiment had a similar value compared with previous studies [1,19,20]. The EE content in the CM was comparable with Lee and Kim [21]. The AA composition of CM was comparable with the composition reported by Sulabo et al [4].

### Amino acid digestibility

There were no differences in the AID and SID of all AA between the NC and PC treatments except that the PC treatment had lower AID and SID of glycine (Gly) than the NC treatment (p<0.05; Table 4, and 5, respectively). The AID of Lys and aspartic acid (Asp) showed linear decreases (p<0.05) with increasing inclusion levels CM in the diets containing β-mannanase. There were linear decreases in SID of Lys (p< 0.05) and of Asp (p = 0.06; tendency) with increasing inclusion rate of CM in the diets containing β-mannanase.

The AID and SID values for AA in the NC treatment agreed with published values [4,22]. No differences in the SID of AA (except for Gly) between the NC and PC treatments indicated that the β-mannanase supplementation did not affect AA digestibility of the corn-SBM based diets. This result could be explained by the fact that β-mannanase only degrades β-mannans as substrates but not protein in the diets. This result agrees with previous studies that reported that the β-mannanase supplementation in corn-SBM based diets for nursery and growing pigs did not affect protein digestibility [7,23]. However, reasons for the reduction in SID of Gly by β-mannanase supplementation is still unclear.

In the current study, reduced SID of Lys and Asp were observed with increasing inclusion rate of CM in the diets containing β-mannanase. In addition, there were numerical decreases in the SID of other AA with increasing level of CM in the diets containing β-mannanase. To our knowledge, no data for SID of AA in the diets containing CM with β-mannanase have been published. Sulabo et al [4] reported that the AID and SID of AA in CM where the range is from 67.6% to 83.5% for AID of AA and from 72.8% to 128.8% for SID of AA, which were significantly lower than corn [24] and SBM [4]. This implies that the addition of CM in corn-SBM based diets could reduce AA digestibility. The CM processing involves several drying steps ranging from 104°C to 110°C for up to 30 min to reduce moisture content to 2% to 3% [4]. Therefore, the heat damage during the CM processing may occur and accelerate Maillard reactions, in turn resulting in reduced AA digestibility [20]. This result agrees with Jang et al [7] who reported that the protein digestibility decreased with increasing inclusion rate of CM in the diets containing β-mannanase for growing pigs.

In conclusion, the results of the current study indicated that the β-mannanase supplementation did affect AA digestibility in pigs and that increasing the inclusion level of CM could reduce SID of AA, particularly Lys and Asp.

## Figures and Tables

**Table 1:** Analyzed chemical and amino acid composition of copra meal (as-fed basis)

Item	%
Nutrient composition	
Dry matter	90.27
Crude protein	22.18
Ether extract	3.84
Calcium	0.62
Phosphorus	0.35
Amino acid composition	
Indispensable amino acids	
Arginine	1.79
Histidine	0.32
Isoleucine	0.64
Leucine	1.23
Lysine	0.42
Methionine	0.21
Phenylalanine	0.68
Threonine	0.60
Valine	0.94
Dispensable amino acids	
Alanine	0.80
Aspartic acid	1.53
Cysteine	0.30
Glutamic acid	3.33
Glycine	0.90
Proline	0.64
Serine	0.73
Tyrosine	0.44

**Table 2:** Diet formulation and chemical composition of experimental diets

Item	Treatment^1)^					N-free diet

	NC	PC	CM6	CM12	CM18	
Ingredients (%)						
Corn	69.16	69.00	63.78	58.60	53.38	-
Soybean meal	28.34	28.36	26.40	24.42	22.46	-
Copra meal	-	-	6.00	12.00	18.00	-
Corn starch	-	-	-	-	-	80.00
Dextrose	-	-	-	-	-	15.00
Soy oil	-	0.04	1.18	2.31	3.45	2.00
Limestone	0.38	0.38	0.42	0.44	0.49	0.50
Tricalcium phosphate	1.11	1.11	1.07	1.04	0.99	1.60
L-lysine∙HCl	0.11	0.11	0.15	0.19	0.23	-
Vitamin mix^2)^	0.10	0.10	0.10	0.10	0.10	0.10
Trace mineral mix^3)^	0.10	0.10	0.10	0.10	0.10	0.10
Salt	0.20	0.20	0.20	0.20	0.20	0.20
Chromic oxide	0.50	0.50	0.50	0.50	0.50	0.50
β-mannanase^4)^	-	0.10	0.10	0.10	0.10	-
Total	100.00	100.00	100.00	100.00	100.00	100.00
Analyzed composition (%)						
Dry matter	91.01	91.77	92.14	91.38	91.04	91.30
Crude protein	18.40	18.38	18.35	18.17	18.04	0.31
Calcium	0.72	0.72	0.71	0.72	0.73	0.49
Phosphorus	0.33	0.32	0.34	0.31	0.44	0.23

CM, copra meal.

1) NC, negative control (corn-soybean meal-based diet); PC, positive control (basal diet + 0.10% β-mannanase, 800 IU/kg diet); CM6, PC diet + 6% CM; CM12, PC diet + 12% CM; CM18, PC diet + 18% CM.

2) Provided per kg of diet: vitamin A, 16,000 IU; vitamin D_3_, 3,200 IU; vitamin E, 35 IU; vitamin K_3_, 5 mg; riboflavin, 6 mg; pantothenic acid, 16 mg; niacin, 32 mg; d-biotin, 128 μg, vitamin B_12_, 20 μg.

3) Provided per kg of diet: Fe, 281 mg; Cu, 288 mg; Mn, 49 mg; I, 0.3 mg; Se, 0.3 mg.

4) CTCzyme: CTC Bio Inc. (Seoul, Korea).

**Table 3:** Analyzed amino acid composition of experimental diets (as-fed basis)

Item	Treatment^1)^				

	NC	PC	CM6	CM12	CM18
Indispensable amino acid (%)					
Arginine	1.22	1.16	1.11	1.18	1.16
Histidine	0.43	0.42	0.39	0.39	0.39
Isoleucine	0.70	0.67	0.61	0.59	0.57
Leucine	1.55	1.54	1.45	1.37	1.56
Lysine	1.11	1.08	0.99	1.01	0.97
Methionine	0.36	0.31	0.27	0.30	0.35
Phenylalanine	0.86	0.84	0.78	0.76	0.78
Threonine	0.75	0.72	0.67	0.67	0.68
Valine	0.75	0.72	0.68	0.68	0.71
Dispensable amino acid (%)					
Alanine	0.89	0.88	0.84	0.83	0.96
Aspartic acid	1.88	1.83	1.69	1.68	1.58
Cysteine	0.30	0.27	0.25	0.24	0.32
Glutamic acid	3.51	3.44	3.25	3.18	3.38
Glycine	0.75	0.73	0.68	0.72	0.72
Proline	0.66	0.68	0.61	0.58	0.68
Serine	0.94	0.92	0.87	0.85	0.89
Tyrosine	0.62	0.59	0.55	0.51	0.51

CM, copra meal.

1) NC, negative control (corn-soybean meal-based diet); PC, positive control (basal diet + 0.10% β-mannanase, 800 IU/kg diet); CM6, PC diet + 6% CM; CM12, PC diet + 12% CM; CM18, PC diet + 18% CM.

**Table 4:** Effect of increasing levels of copra meal with β-mannanase on apparent ileal digestibility of amino acids in pigs^1)^

Item	Treatment^2)^					SEM	p-values^3)^		
	
	NC	PC	CM6	CM12	CM18		NC vs PC	Lin	Quad
Indispensable amino acid (%)									
Arginine	93.38	92.24	92.01	92.65	91.90	0.396	0.18	0.85	0.57
Histidine	91.14	90.19	90.40	89.88	87.53	1.353	0.50	0.23	0.41
Isoleucine	89.16	87.84	88.21	87.99	84.72	1.284	0.42	0.13	0.20
Leucine	89.86	89.15	90.37	90.11	90.60	0.655	0.59	0.11	0.50
Lysine	90.00	88.97	88.47	86.09	85.29	0.872	0.47	0.01	0.88
Methionine	92.58	92.32	90.59	91.21	92.09	1.146	0.90	0.99	0.20
Phenylalanine	90.05	89.39	89.70	89.92	89.32	0.533	0.59	0.99	0.47
Threonine	84.30	81.38	82.77	82.16	80.44	1.039	0.25	0.53	0.22
Valine	85.44	82.39	83.86	83.26	82.17	1.224	0.26	0.84	0.36
Dispensable amino acid (%)									
Alanine	83.35	81.22	82.93	81.79	83.56	1.210	0.37	0.28	0.98
Aspartic acid	87.75	86.89	86.60	83.89	83.15	0.855	0.57	0.02	0.27
Cysteine	85.53	84.16	81.41	83.97	83.84	3.076	0.44	0.92	0.70
Glutamic acid	89.69	90.87	87.93	87.70	89.20	0.843	0.15	0.22	0.11
Glycine	81.67	76.13	74.43	74.09	75.97	1.883	0.01	0.92	0.32
Proline	85.02	84.35	81.23	84.41	83.19	1.970	0.86	0.97	0.65
Serine	87.25	86.10	86.84	85.87	84.60	0.974	0.47	0.26	0.35
Tyrosine	89.21	88.46	88.79	87.88	85.68	1.150	0.65	0.12	0.32

CM, copra meal; SEM, standard error of the means.

1) n = 4 per treatment.

2) NC, negative control (corn-soybean meal-based diet); PC, positive control (basal diet + 0.10% β-mannanase, 800 IU/kg diet); CM6, PC diet + 6% CM; CM12, PC diet + 12% CM; CM18, PC diet + 18% CM.

3) p-values are for the single degree of freedom contrast between NC and PC treatments and linear and quadratic responses based on CM supplementation levels with PC, CM6, CM12, and CM18 treatments.

**Table 5:** Effect of increasing levels of copra meal with β-mannanase on standardized ileal digestibility of amino acids in pigs1)

Item	Treatment^2)^					SEM	p-values^3)^		
	
	NC	PC	CM6	CM12	CM18		NC vs PC	Lin	Quad
Indispensable amino acid (%)									
Arginine	98.46	97.59	97.59	97.90	97.27	0.420	0.29	0.74	0.49
Histidine	96.02	95.19	95.78	95.27	92.92	1.344	0.55	0.29	0.34
Isoleucine	95.58	94.56	95.59	95.62	92.61	1.195	0.52	0.35	0.16
Leucine	93.66	92.98	94.44	94.42	94.38	0.606	0.61	0.15	0.18
Lysine	93.51	92.58	92.41	89.95	89.31	0.835	0.51	0.02	0.81
Methionine	98.69	99.41	98.74	98.54	98.46	1.166	0.74	0.49	0.76
Phenylalanine	93.88	93.32	93.93	94.27	93.55	0.613	0.64	0.71	0.30
Threonine	91.90	89.30	91.28	90.66	88.88	0.988	0.30	0.73	0.14
Valine	93.70	91.00	92.97	92.38	90.97	1.224	0.31	0.91	0.23
Dispensable amino acid (%)									
Alanine	90.99	88.95	91.03	89.98	90.64	0.990	0.39	0.45	0.56
Aspartic acid	92.75	92.03	92.16	91.40	89.12	0.873	0.63	0.06	0.25
Cysteine	94.86	94.53	92.61	95.64	92.73	3.081	0.85	0.87	0.88
Glutamic acid	93.31	94.56	91.84	91.70	92.97	0.844	0.13	0.25	0.15
Glycine	95.27	90.10	89.43	88.26	90.14	1.602	0.01	0.89	0.47
Proline	111.83	110.38	110.25	114.92	109.41	1.942	0.70	0.85	0.21
Serine	94.16	93.16	94.31	93.51	91.94	0.928	0.53	0.35	0.21
Tyrosine	94.70	94.22	94.97	94.55	92.35	0.990	0.77	0.29	0.25

CM, copra meal; SEM, standard error of the means.

1) n = 4 per treatment.

2) NC, negative control (corn-soybean meal-based diet); PC, positive control (basal diet + 0.10% β-mannanase, 800 IU/kg diet); CM6, PC diet + 6% CM; CM12, PC diet + 12% CM; CM18, PC diet + 18% CM.

3) p-values are for the single degree of freedom contrast between NC and PC treatments and linear and quadratic responses based on CM supplementation levels with PC, CM6, CM12, and CM18 treatments.
